# Correction to ‘Rare ribosomal RNA sequences from archaea stabilize the bacterial ribosome’

**DOI:** 10.1093/nar/gkae047

**Published:** 2024-01-23

*Nucleic Acids Research*, Volume 51, Issue 4, 28 February 2023, Pages 1880–1894, https://doi.org/10.1093/nar/gkac1273

Since publication, the authors have discovered unexpected background mutations in two of the plasmids used in the work.

In the plasmids encoding the A2451C mutation in 23S rRNA, the authors discovered the background mutations nearly inactivated those ribosomes, both with the wild-type and CC mutation in the A loop. When these background mutations are corrected, both ribosomes are in fact active. Although the CC mutation in the A loop leads to slightly lower activity of A2451C ribosomes at 37°C, the authors still find the CC mutation in the A loop increases the thermostability of ribosomes with the A2451C mutation compared to the A2451C control with a WT A loop (Supplementary Data).

The authors think the background mutations developed because the A2451C mutation is a dominant lethal mutation. Some leaky expression during plasmid propagation probably led to toxicity in cells that was ‘solved’ by inactivating the mutant ribosomes.

The authors have sequenced all the plasmids used in the work using an intact plasmid sequencing service, and the two plasmids described above were the only two affected.

The results and conclusion of the article are not affected and remain valid.

## Supplementary Material

gkae047_Supplemental_File

