# Supplementary material for: Rare ribosomal RNA sequences from archaea stabilize the bacterial ribosome
Source: Nucleic Acids Res. 2023 Jan 20;51(4):1880–94. doi: 10.1093/nar/gkac1273 (PMC9976906; doi:10.1093/nar/gkac1273)
Supplement: gkac1273_Supplemental_File [file gkac1273_supplemental_file.pdf]

Supplementary Data for

Rare Ribosomal RNA Sequences from Archaea Stabilize the Bacterial Ribosome

Amos J. Nissley[1], Petar I. Penev[2], Zoe L. Watson[3], Jillian F. Banfield[2,4,5], Jamie H. D. Cate[1,2,3,6,7]*

[1] Department of Chemistry, University of California, Berkeley, Berkeley, California, 94720, United States
[2] Innovative Genomics Institute, University of California, Berkeley, Berkeley, California, 94704, United States
[3] California Institute for Quantitative Biosciences, University of California, Berkeley, Berkeley, California, 94720, United States
[4] Earth and Planetary Science, University of California, Berkeley, Berkeley, California, 94720, United States
[5] Environmental Science, University of California, Berkeley, Berkeley, California, 94720, United States
[6] Department of Molecular and Cell Biology, University of California, Berkeley, Berkeley, California, 94720, United States
[7] Molecular Biophysics and Integrated Bioimaging Division, Lawrence Berkeley National Laboratory, Berkeley, California, 94720, United States

* To whom correspondence should be addressed. Tel: +15106662749; Fax: +15106662747; Email: j-h-doudna-cate@berkeley.edu

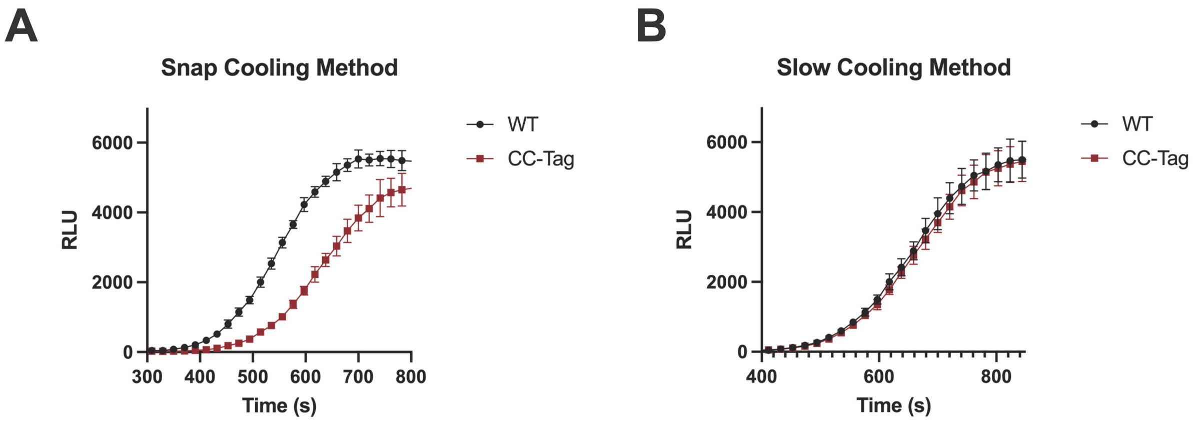

Figure S1. HiBit translation assay for untagged WT and MS2-tagged CC 50S ribosomes. Ribosomes were pre-incubated at 50 °C for 30 minutes and then cooled quickly on ice (left) or allowed to cool more slowly by incubating at room temperature for an additional 30 minutes (right).

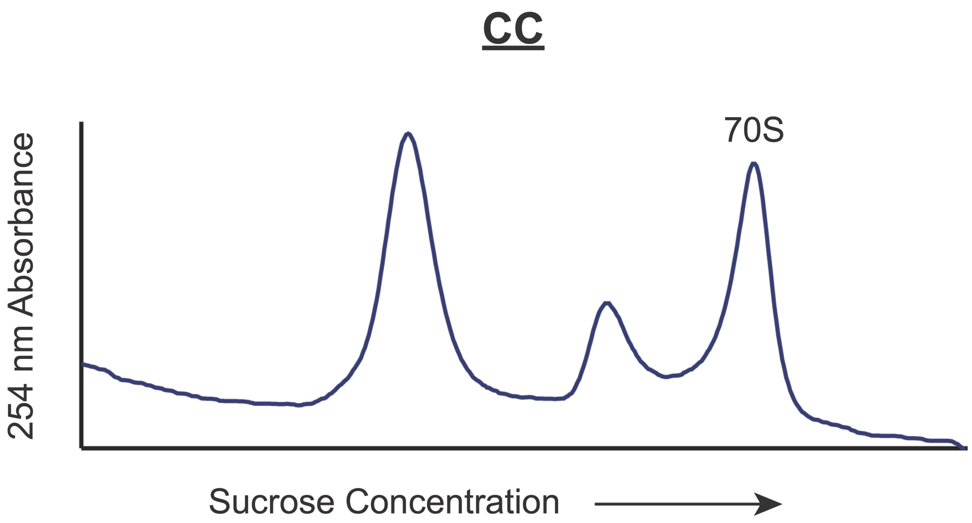

Figure S2. Association gradient of CC mutant 50S and WT *E. coli* 30S subunits. 30S subunits (2 equiv.) and 50S subunits (1equiv.) were incubated with 10 mM $MgCl_2$ and resolved on a 15-40% sucrose gradient. 70S fractions were collected for cryo-EM analysis.

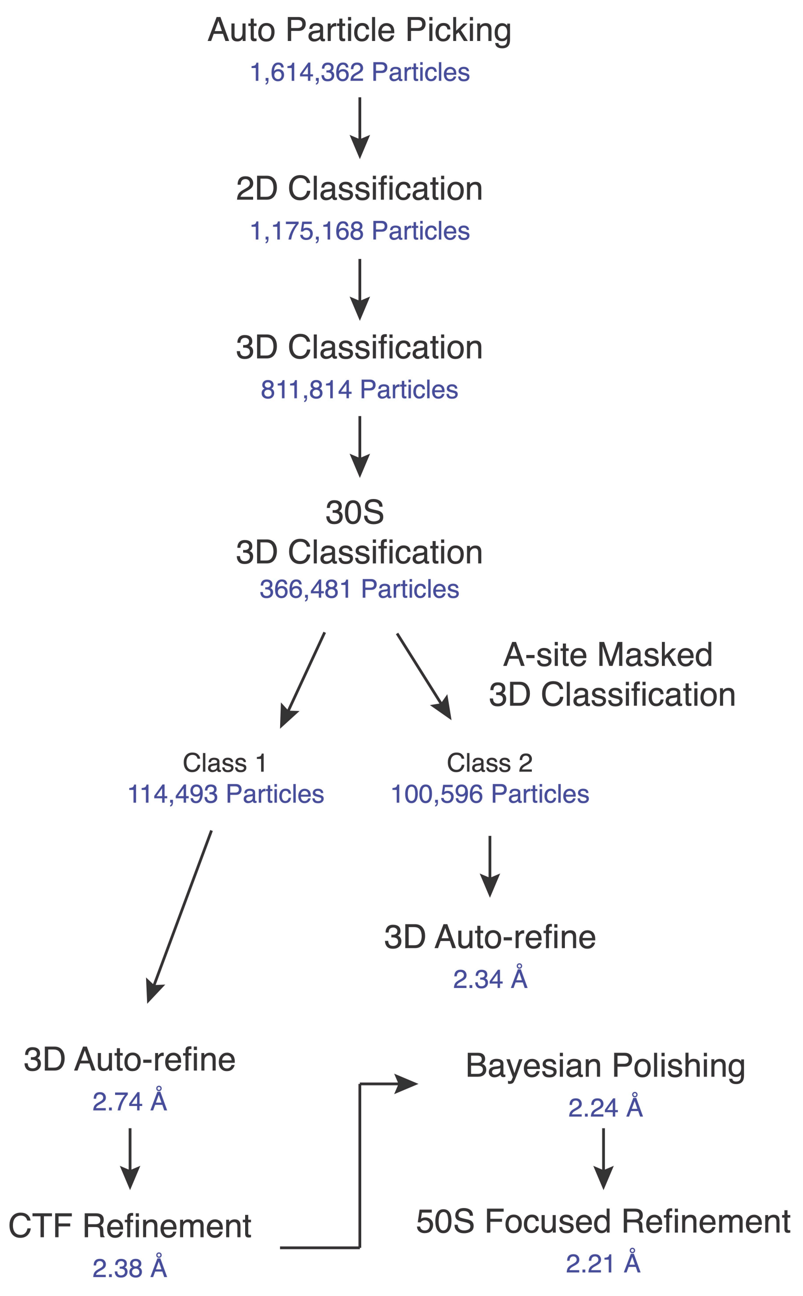

Figure S3. Cryo-EM processing workflow. A-site Class 1 had density for the tRNA 3´-CCA end and was further refined. Class 2 lacked density for the A-site tRNA 3´-CCA end.

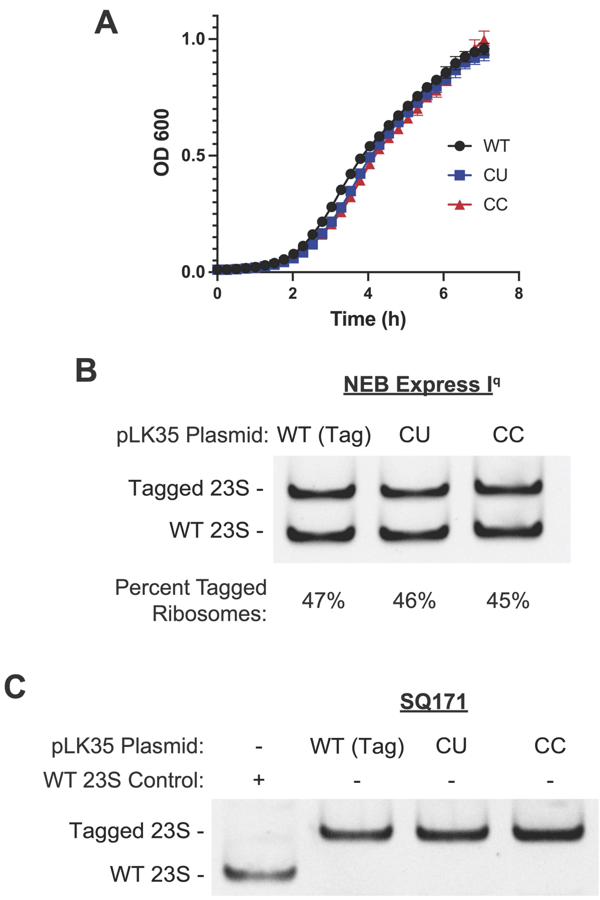

Figure S4. Effects of Mutant A loop Sequences on *E. Coli* Growth. A) Growth curves of *E. coli* Express I^q cells which express both plasmid encoded and genomically encoded ribosomes (data is represented as the mean of 3 replicates). Error bars represent the standard deviation of three independent replicates. The plasmid-encoded 23S rRNA harbors an MS2 tag. B) Following *E. coli* growth assays, the ratio of MS2-tagged ribosomes to WT endogenous ribosomes was determined. RT-PCR samples were run on a 10% polyacrylamide-TBE gel. DNA containing an MS2 tag sequence is 32 bp larger than DNA with the native 23S sequence. Amplified bands were quantified to determine the percent of MS2-tagged ribosomes. C) Ribosomes from *E. coli* strain SQ171 were subjected to RT-PCR to confirm that only plasmid encoded (MS2 tag) ribosomes were expressed during growth experiments (**Figure 2A**).

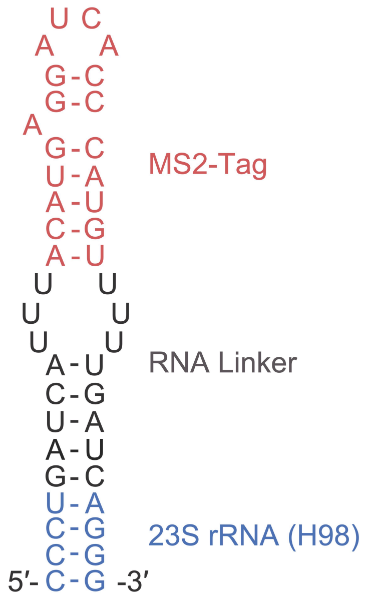

Figure S5. MS2-Tag design used in this study. The MS2 tag is inserted into rRNA helix H98 in the 50S *E. coli* ribosome. The H98 stem is elongated and the MS2 stem loop is attached with a poly-U linker.

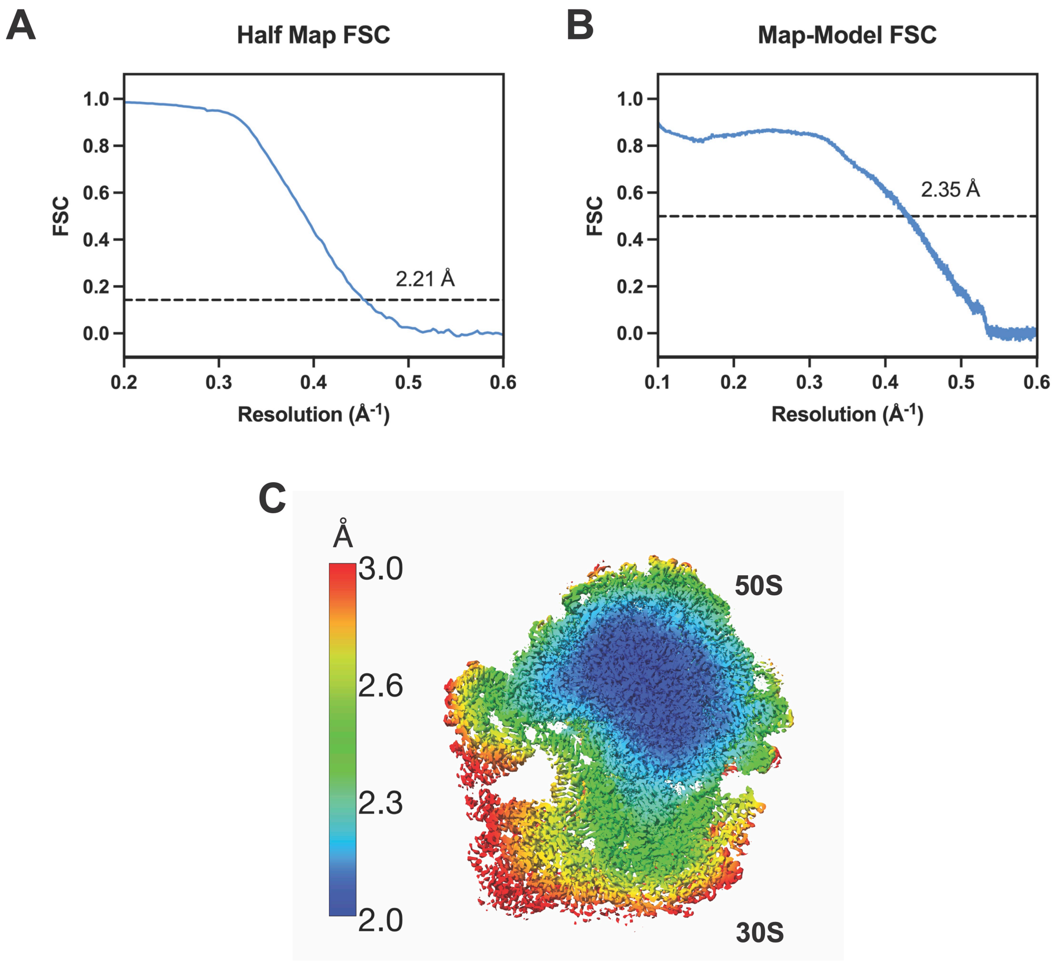

Figure S6. Resolution of the cryo-EM map generated from the particles in Class I. The global 70S ribosome A) half map FSC resolution is 2.21 Å and B) map to model FSC resolution is 2.35 Å. C) Local resolution is plotted on the 70S ribosome map.

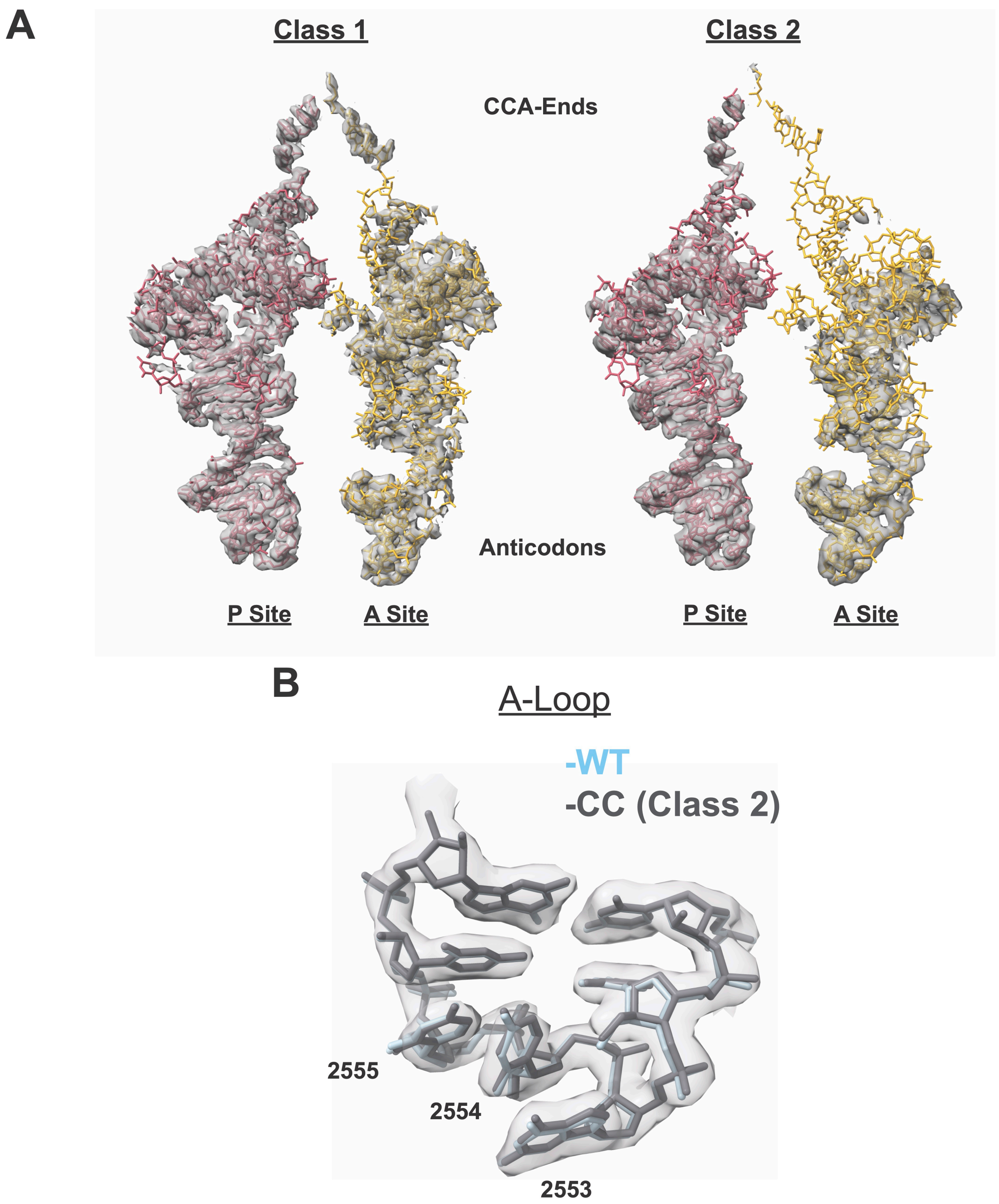

Figure S7. Features of the tRNAs in A-site classes 1 and 2. A) P-site and A-site tRNA model and cryo-EM density for A-site class 1 (left) and class 2 (right). Class 2 corresponded to an A-site tRNA class that lacked density for most of the acceptor stem. A B-factor of 30 Å^2 was applied to the cryo-EM maps. B) Cryo-EM density and model for the A-loop from Class 2 are shown in grey. The model from a WT ribosome structure (1) is shown in light blue.

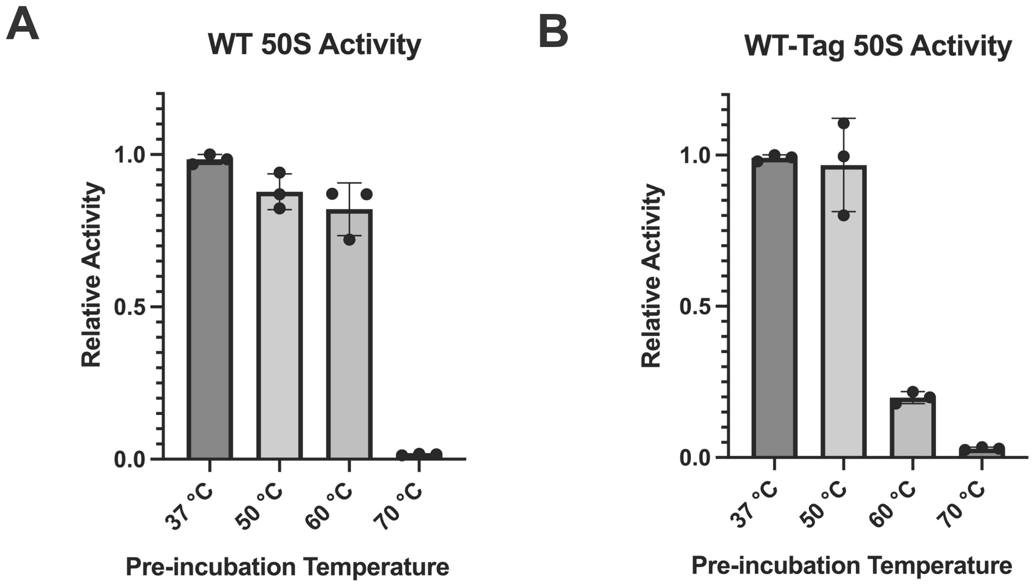

Figure S8. Activity of heat-treated 50S subunits without and with the MS2 tag. A) Untagged and B) MS2-tagged WT 50S ribosomes were pre-incubated at the indicated temperatures for 30 minutes and then slow cooled for 30 minutes. The 50S ribosomes were then used in the HiBit translation assay (data is represented as the mean of 3 replicates). Error bars represent the standard deviation of three reactions.

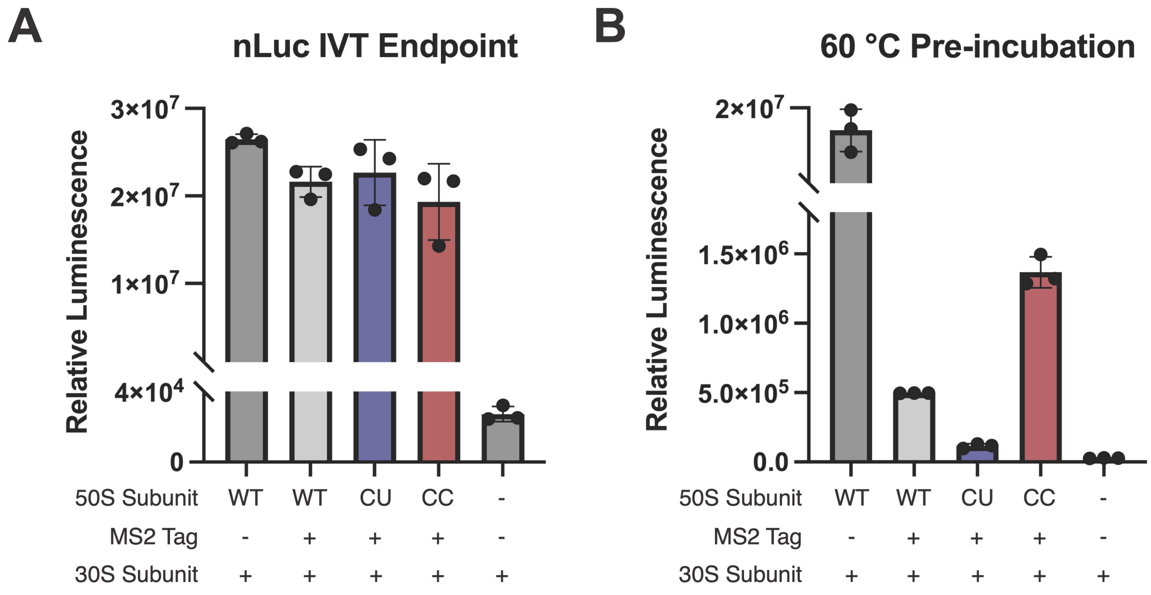

Figure S9. Activity of ribosomes in nLuc translation endpoint assays. A) Replicate of an nLuc IVT endpoint assay at 37 °C for CU and CC ribosomes. B) 50S ribosomes were preincubated at 60 °C and cooled slowly. Their relative activities were then determined with the nLuc endpoint assay.

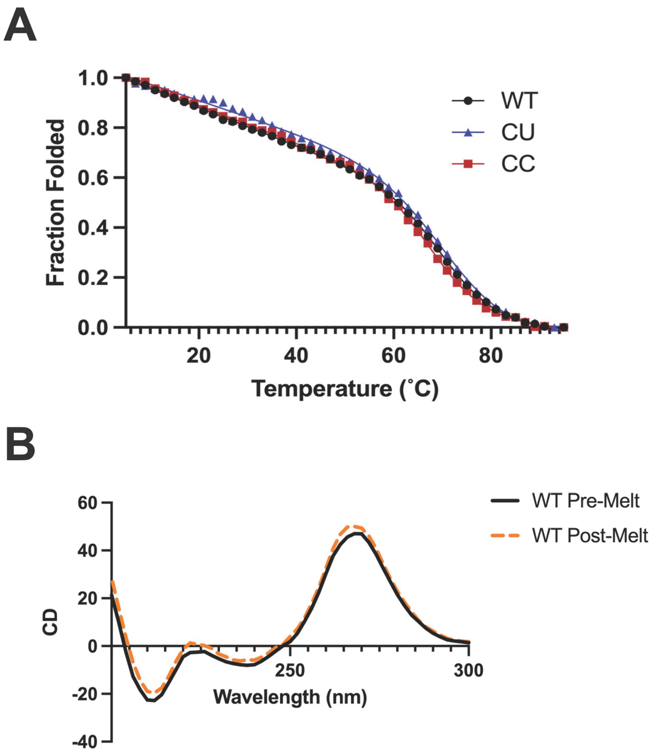

Figure S10. CD unfolding of A-loop RNA constructs. A) Full CD unfolding data from 5 to 95 °C. RNA constructs demonstrated cooperative two-state unfolding. B) CD wavelength scans from 300 to 200 nm of the WT A-loop construct before the melting experiment (black) and after the melting experiment (orange). The similarity of the curves implies reversible unfolding of the RNA during melting experiments.

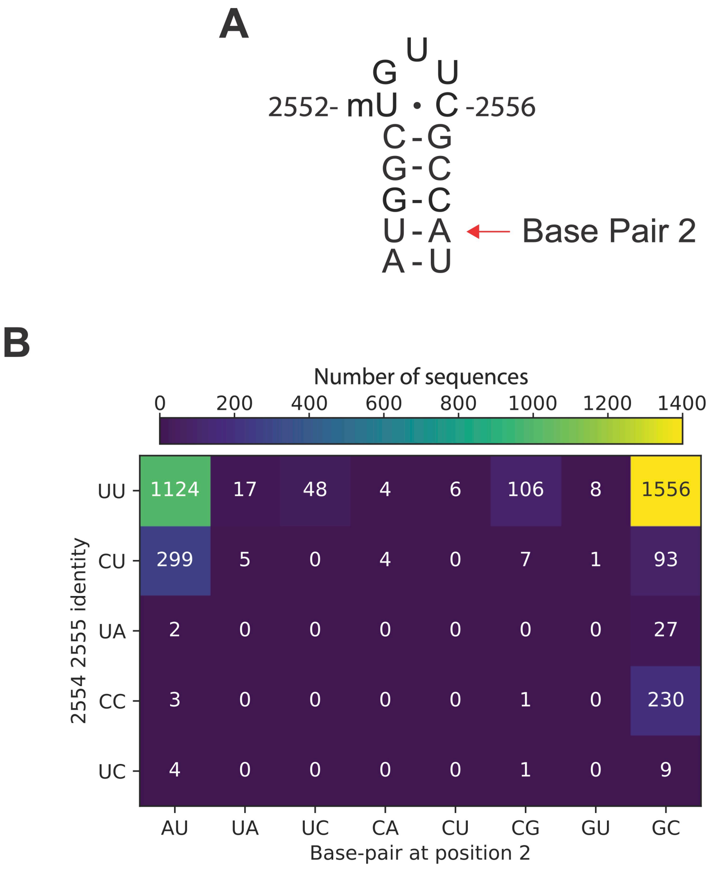

Figure S11. Archaeal sequences at the base of their A-loop. A) Base pair 2 (2548-2560) is highlighted on the secondary structure of the *E. coli* A-loop. B) Distribution of archaeal sequences in the SILVA database based on 2554/2555 and base pair 2 identity. The left axis classifies Archaea based on the identity of nucleotides at positions 2554 and 2555. The main populations are UU, CU, and CC. The bottom axis classifies Archaea based on the nucleotide identity at base pair 2 in the A-loop. Almost all Archaea with cytidines at positions 2554 and 2555 have a GC base pair at position 2. Around half of Archaea with uridines at positions 2554 and 2555 also have a GC base pair at position 2.

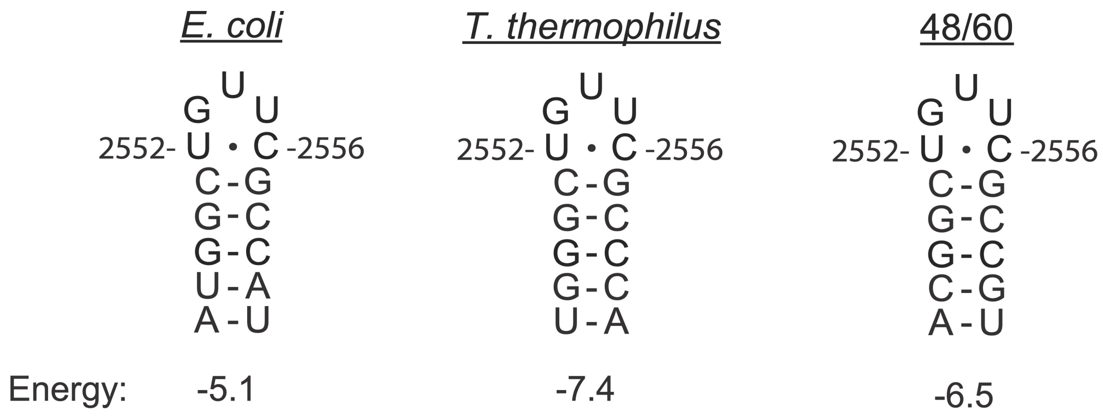

Figure S12. Secondary structures and predicted minimum free energies of A-loop mutants. *T. thermophilus* has variation at the base of the A-loop that stabilize its secondary structure. The 48/60 mutant was designed to stabilize the base of the A-loop while leaving pyrimidine bases at positions 2548 and 2561 to maintain contacts with ribosomal protein uL14. Relative minimum free energies were calculated in RNAstructure (2).

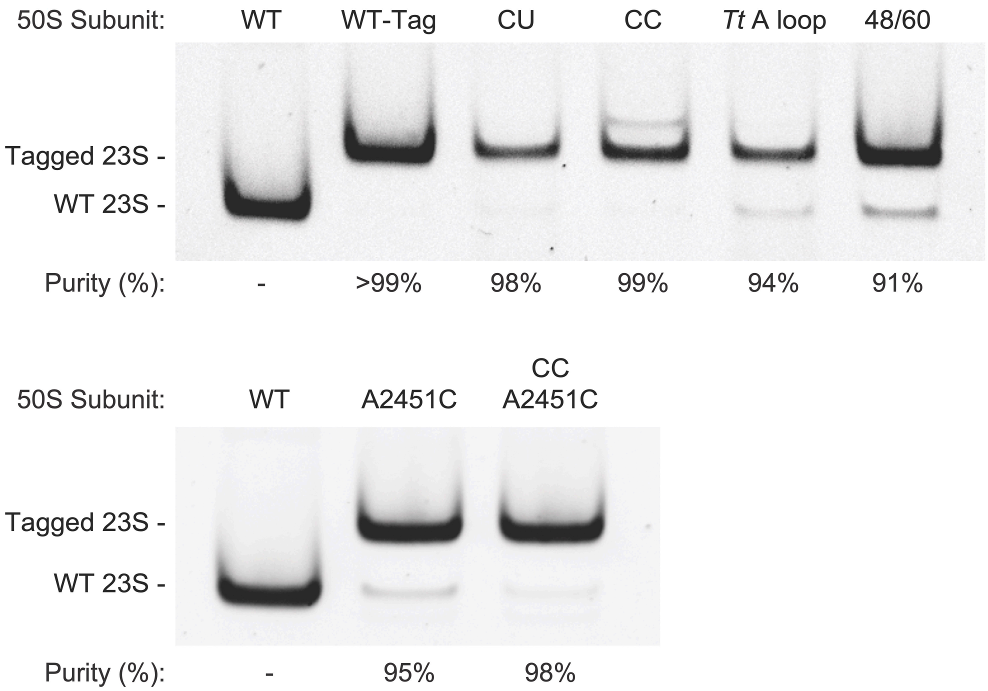

Figure S13. Purity of 50S subunits used for *in vitro* assays. After MS2 purification, 23S rRNA was isolated from 50S subunits and used for RT-PCR analysis. DNA containing an MS2 tag sequence is 32 bp larger than DNA with the native 23S sequence. RT-PCR products were run on a 10% polyacrylamide-TBE gel, and band intensities were used to quantify ribosome purity.

Table S1. Additional Growth Temperatures for Archaeal Taxa

Name	Strain	TaxID	Taxonomy	Growth Temperature	Source
Nitrosopelagicus brevis	CN25	1410606	Archaea;Thaumarchaeota	22 °C	(3)
Nitrosotalea devanaterra	-	1078905	Archaea;Thaumarchaeota	25 °C	(4)
Nitrosotenuis aquarius	-	1846278	Archaea;Thaumarchaeota	33 °C	(5)
Nitrosocaldus islandicus	-	1846278	Archaea;Thaumarchaeota; Nitrososphaeria	65 °C	(6)
Nitrosocosmicus franklandus	C13	1798806	Archaea;Thaumarchaeota; Nitrososphaeria; Nitrososphaerales; Nitrososphaeraceae	37.5 °C	(7)
Caldiarchaeum subterraneum	-	1798806	Archaea;Thaumarchaeota	70 °C	(8)

Table S2. DNA primers used in this study
(m denotes 2'-O-Me; lowercases in the sequence indicate sites where mutations are introduced)

**Primer name**	**Sequence**	**Comment**
U2554C_F	GGTATGGCTGcTCGCCATTTA	Forward primer for U2554C 23S rRNA mutagenesis
U2554C_R	CTTGGGACCTACTTCAGC	Reverse primer for U2554C 23S rRNA mutagenesis
U2554/5C_F	GGTATGGCTGccCGCCATTTAAAG	Forward primer for U2554C U2555C 23S rRNA mutagenesis
U2554/5C_R	CTTGGGACCTACTTCAGC	Reverse primer for U2554C U2555C 23S rRNA mutagenesis
A2058G_F	GGCAAGACGGgAAGACCCCGT	Forward primer for A2058G 23S rRNA mutagenesis
A2058G_R	GCGGGTACACTGCATCTTC	Reverse primer for A2058G 23S rRNA mutagenesis
Tt_Aloop_F	TCGCCcaTTAAAGTGGTACGCGAGC	Forward primer for A2547U U2548G A2560C U2561A 23S rRNA mutagenesis (*Tt* A loop)
Tt_Aloop_R	ACAGCCcaACCCTTGGGACCTACTTC	Reverse primer for A2547U U2548G A2560C U2561A 23S rRNA mutagenesis (*Tt* A loop)
48/60_Aloop_F	TCGCCgTTTAAAGTGGTACGCGAG	Forward primer for U2548C A2560G 23S rRNA mutagenesis (48/60)
48/60_Aloop_R	ACAGCCgTACCCTTGGGACCTACTT	Reverse primer for U2548C A2560G 23S rRNA mutagenesis (48/60)
A2451C_F	TCCGGGGATAcCAGGCTGATA	Forward primer for A2451C 23S rRNA mutagenesis
A2451C_R	GTACCTTTTATCCGTTGAGC	Reverse primer for A2451C 23S rRNA mutagenesis
MS2_quant_F	CTTGCCCCGAGATGAGTTCTCCC	RT-PCR primer for the ribosome purity assay
MS2_quant_R	GTACCGGTTAGCTCAACGCATCGCT	Primer for amplification of cDNA in the ribosome purity assay
HiBit Template	GCGAATTAATACGACTCACTATAGGGTTAACTTT AACAAGGAGAAAAACATGGTGAGCGGCTGGCG CCTGTTTAAAAAAATTAGCTAACTAGCATAACCC CTCTCTAAACGGAGGGGTTTAGTCA	DNA template for the HiBit peptide
HiBit_amp_F	GCGAATTAATACGACTCACTATAG	Forward amplification primer for the HiBit template
HiBit_amp_R	AAACCCCTCCGTTTAGAG	Reverse amplification primer for the HiBit template
tRNA^{fMET} Template (C1G)	AATTCCTGCAGTAATACGACTCACTATAGGCGG GGTGGAGCAGCCTGGTAGCTCGTCGGGCTCAT	DNA template for tRNA^{fMet} C1G - A

	AACCCGAAGGTCGTCGGTTCAAATCCGGCCCC CGCAACC	
fMet_amp_F	AATTCCTGCAGTAATACGACTCAC	Forward amplification primer for the $tRNA^{fMet}$ template
fMet-A_amp_R	mGmGTTGCGGGGGCC	Reverse amplification primer for the $tRNA^{fMet}$ template

Table S3. RNA oligos (IDT) used in this study. (m denotes 2'-*O*-Me)

**Name**	**Sequence**
WT A loop	AUGGCmUGUUCGCCAU
CU A loop	AUGGCmUGCUCGCCAU
CC A loop	AUGGCmUGCCCGCCAU

Table S4. Cryo-EM Data Collection and Processing

Magnification	105,000
Voltage (kV)	300
Electron Exposure ($e^-/Å^2$)	40
Defocus Range (μm)	-0.5/-1.5
Pixel Size (Å)	0.8279
Symmetry Imposed	C1
Initial Particle Images	1,614,362
Final Particle Images	114,493
Map Resolution (Å)	2.21
FSC Threshold	0.143

Table S5. Model Refinement Statistics

Model component	
Model resolution (Å)	2.35
FSC threshold	0.5
Map sharpening *B* factor (Å^2)	-38.8
Model composition	
Non-hydrogen atoms	148866
Mg^{2+} ions	268
Zn^{2+} ions	2
Polyamines	17
Waters	6756
Ligands (paromomycin)	1
Mean *B* Factors (Å^2)	
RNA	15.98
Protein	15.81
Waters	8.73
Other	10.56
R.m.s. deviations from ideal values	
Bond (Å)	0.005
Angle (°)	0.762
Molprobity all-atom clash score	8.93
Ramachandran plot	
Favored (%)	96.37
Allowed (%)	3.58
Outliers (%)	0.05
RNA validation	
Angles outliers (%)	0.004
Sugar pucker outliers (%)	0.2
Average suiteness	0.587

Table S6. Doubling times for *E. coli* expressing plasmid encoded ribosome mutants

E. coli **Strain**	**pLK35 Plasmid Sequence**	**Doubling Time (m)**
NEB Express I^q	WT	36.0 ± 0.6
NEB Express I^q	CU	35.7 ± 0.3
NEB Express I^q	CC	38.4 ± 0.6
SQ171	WT	60 ± 1
SQ171	CU	66 ± 3
SQ171	CC	61 ± 2

Table S7. Thermodynamic parameters derived from CD melting experiments for A-loop RNA constructs.[+]

Construct	T_m (° C)	ΔH_{unfold} (kJ/mol)	ΔG_{25}[++] (kJ/mol)
WT	71.8 ± 0.6	197 ± 9	26.8 ± 0.9
CU	71.8 ± 0.1	189 ± 2	25.7 ± 0.3
CC	69.0 ± 0.2	183 ± 8	23.6 ± 1.2

+ Errors are reported as the S.D. of two experimental replicates.
++ ΔG_{25} is the Gibbs free energy of unfolding at 25 °C.

References

1. Watson,Z.L., Knudson,I., Ward,F.R., Miller,S.J., Cate,J.H.D., Schepartz,A. and Abramyan,A.M. (2022) Atomistic simulations of the E. coli ribosome provide selection criteria for translationally active substrates. *biorXiv*, doi: https://doi.org/10.1101/2022.08.13.503842, 13 August 2022, pre-print: not peer-reviewed.
2. Bellaousov,S., Reuter,J.S., Seetin,M.G. and Mathews,D.H. (2013) RNAstructure: Web servers for RNA secondary structure prediction and analysis. *Nucleic Acids Res.*, **41**, 471–474.
3. Santoro,A.E., Dupont,C.L., Richter,R.A., Craig,M.T., Carini,P., McIlvin,M.R., Yang,Y., Orsi,W.D., Moran,D.M. and Saito,M.A. (2015) Genomic and proteomic characterization of 'Candidatus Nitrosopelagicus brevis': An ammonia-oxidizing archaeon from the open ocean. *Proc. Natl. Acad. Sci. USA*, **112**, 1173–1178.
4. Lehtovirta-Morley,L.E., Stoecker,K., Vilcinskas,A., Prosser,J.I. and Nicol,G.W. (2011) Cultivation of an obligate acidophilic ammonia oxidizer from a nitrifying acid soil. *Proc. Natl. Acad. Sci. USA*, **108**, 15892–15897.
5. Sauder,L.A., Engel,K., Lo,C., Chain,P. and Neufeld,J.D. (2018) "Candidatus Nitrosotenuis aquarius," an Ammonia-Oxidizing Archaeon from a Freshwater Aquarium Biofilte. *Appl. Enviromental Microbiol.*, **84**.
6. Daebeler,A., Herbold,C.W., Vierheilig,J., Sedlacek,C.J., Pjevac,P., Albertsen,M., Kirkegaard,R.H., de la Torre,J.R., Daims,H. and Wagner,M. (2018) Cultivation and genomic analysis of 'Candidatus Nitrosocaldus islandicus,' an obligately thermophilic, ammonia-oxidizing thaumarchaeon from a hot spring biofilm in Graendalur valley, Iceland. *Front. Microbiol.*, **9**, 1–16.
7. Lehtovirta-Morley,L.E., Ross,J., Hink,L., Weber,E.B., Gubry-Rangin,C., Thion,C., Prosser,J.I. and Nicol,G.W. (2016) Isolation of 'Candidatus Nitrosocosmicus franklandus', a novel ureolytic soil archaeal ammonia oxidiser with tolerance to high ammonia concentration. *FEMS Microbiol. Ecol.*, **92**, 1–10.
8. Beam,J.P., Jay,Z.J., Schmid,M.C., Rusch,D.B., Romine,M.F., M Jennings,R. De, Kozubal,M.A., Tringe,S.G., Wagner,M. and Inskeep,W.P. (2016) Ecophysiology of an uncultivated lineage of Aigarchaeota from an oxic, hot spring filamentous 'streamer' community. *ISME J.*, **10**, 210–224.
