# Supplementary material for: Correction to ‘Rare ribosomal RNA sequences from archaea stabilize the bacterial ribosome’
Source: Nucleic Acids Res. 2024 Jan 23;52(4):2092. doi: 10.1093/nar/gkae047 (PMC10899748; doi:10.1093/nar/gkae047)
Supplement: gkae047_Supplemental_File [file gkae047_supplemental_file.pdf]

Rare Ribosomal RNA Sequences from Archaea Stabilize the Bacterial Ribosome

Amos J. Nissley[1], Petar I. Penev[2], Zoe L. Watson[3], Jillian F. Banfield[2,4,5], Jamie H. D. Cate[1,2,3,6,7]*

[1] Department of Chemistry, University of California, Berkeley, Berkeley, California, 94720, United States
[2] Innovative Genomics Institute, University of California, Berkeley, Berkeley, California, 94704, United States
[3] California Institute for Quantitative Biosciences, University of California, Berkeley, Berkeley, California, 94720, United States
[4] Earth and Planetary Science, University of California, Berkeley, Berkeley, California, 94720, United States
[5] Environmental Science, University of California, Berkeley, Berkeley, California, 94720, United States
[6] Department of Molecular and Cell Biology, University of California, Berkeley, Berkeley, California, 94720, United States
[7] Molecular Biophysics and Integrated Bioimaging Division, Lawrence Berkeley National Laboratory, Berkeley, California, 94720, United States

* To whom correspondence should be addressed. Tel: +15106662749; Fax: +15106662747; Email: j-h-doudna-cate@berkeley.edu

After publication, the authors became aware of errors in the sequence of two plasmids used in this study. The 23S rRNA gene for plasmids with A2451C and CC-A2451C (U2554C-U2555C-A2451C) subunits each contained an additional point mutation, C2064A and G977T respectively. These unintended mutations were outside of the region that was sequenced with Sanger sequencing during the original study. All other plasmids used in the study were subsequently inspected with nanopore sequencing (Plasmidsaurus) and have the full correct sequences.

Plasmids containing the A2451C and CC-A2451C mutations were cloned into the original expression plasmid and confirmed to have the full correct sequence using nanopore sequencing (Plasmidsaurus). After expression of these subunits, the experiments in **Figure 5** were repeated. We found that the ribosomes with an A2451C mutation in the 23S rRNA have roughly 55% of WT activity and are not inactivated. We additionally find that subunits with CC-A2451C mutations are slightly less active than subunits with an A2451C mutation. However, after heat treatment at 60 °C, CC-A2451C subunits are more active than A2451C subunits and have roughly the same activity as WT subunits with an MS2 tag. This indicates that while the CC mutations do not increase the activity of A2451C subunits at 37 °C, they stabilize the A2451C 50S subunit. Additionally, we find that both A2451C and CC A2451C subunits form 70S subunits, in line with *in vitro* translation assays.

These results indicate that 23S rRNA mutations at positions 977 or 2064 in combination with an A2451C mutation inactivate the 50S subunit. An A2451C mutation in the *E. coli* ribosome was shown to be dominant lethal (1), and the additional inactivating mutations in the 23S rRNA were likely selected as these mutations would negate the toxicity of this gene during cloning. Since subunits with an A2451C mutation retain activity and can form 70S ribosomes we no longer expect the CC mutations to stabilize this ribosome at 37 °C. However, we show that the CC mutations increase the thermostability of the A2451C subunit. While these data affect our interpretation of the effects of CC mutations on the A2451C mutant ribosome, they do not affect our original conclusions that CC mutations stabilize WT and mutant *E. coli* ribosomes.

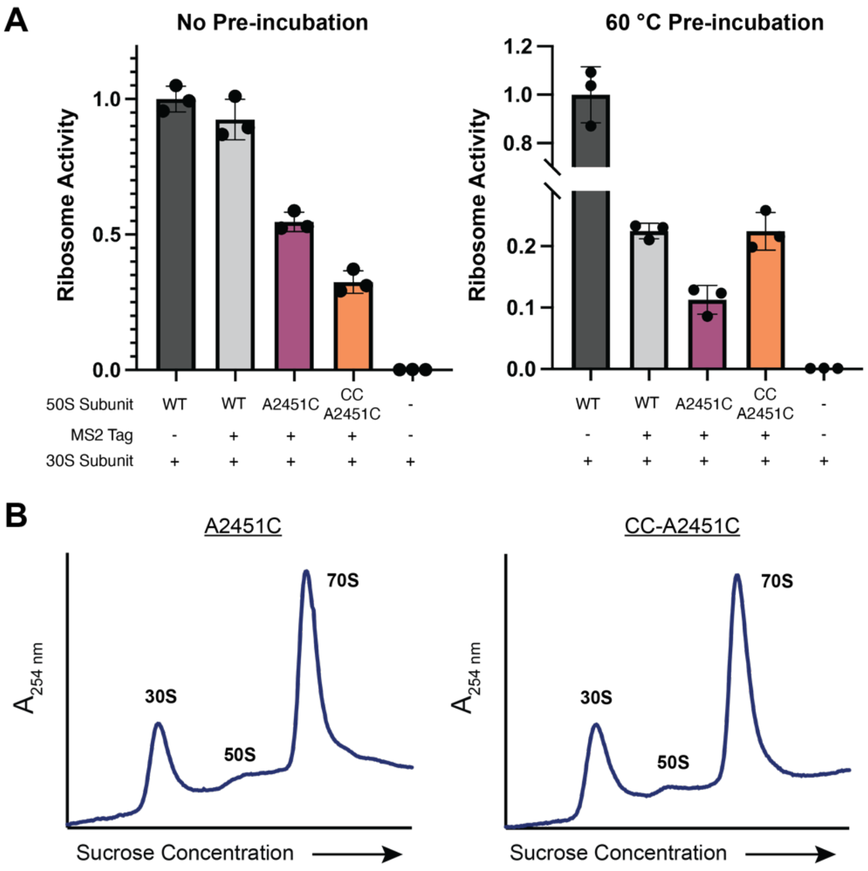

Figure 5. Robustness of the PTC in CC ribosomes. A) The relative activities of 50S subunits with an A2451C mutation or the triple mutation CC-A2451C were determined using the HiBit assay (data is represented as the mean of 3 replicates). Subunits were added directly (left) or pre-incubated at 60 °C (right) before addition to the HiBit assay. Error bars represent the standard deviation of three independent reactions. C) 70S formation by A2451C (left) and CC-A2451C 50S subunits (right).

References

1. Thompson,J., Kim,D.F., O'Connor,M., Lieberman,K.R., Bayfield,M.A., Gregory,S.T., Green,R., Noller,H.F. and Dahlberg,A.E. (2001) Analysis of mutations at residues A2451 and G2447 of 23S rRNA in the peptidyltransferase active site of the 50S ribosomal subunit. *Proc. Natl. Acad. Sci.*, **98**, 9002–9007.
